# In Reply: Low and Borderline Ankle-Brachial Index Is Associated With Intracranial Aneurysms: A Retrospective Cohort Study

**DOI:** 10.1227/neu.0000000000003059

**Published:** 2024-06-13

**Authors:** Dan Laukka, Essi Kangas, Aino Kuusela, Jussi Hirvonen, Tiia Rissanen, Melissa Rahi, Juri Kivelev, Ville Rantasalo, Maarit Venermo, Jaakko Rinne, Harri Hakovirta

**Affiliations:** * Department of Neurosurgery, Neurocenter, Turku University Hospital, Turku, Finland;; ‡ Clinical Neurosciences, University of Turku, Turku, Finland;; § Department of Surgery, University of Turku, Turku, Finland;; ‖ Department of Radiology, Turku University Hospital and University of Turku, Turku, Finland;; ¶ Department of Radiology, University of Tampere, Tampere, Finland;; # Department of Clinical Medicine, Biostatistics, University of Turku, Turku, Finland;; ** Department of Biostatistics, University of Turku and Turku University Hospital, Turku, Finland;; †† Department of Vascular Surgery, University of Helsinki and Helsinki University Hospital, Helsinki, Finland;; ‡‡ Department of Surgery, Satasairaala, Pori, Finland

To the Editor:

We would like to express our gratitude to Oliveira et al^1^ for taking the time to provide their insightful comments on our recently published article entitled “Low and Borderline Ankle–Brachial Index Is Associated With Intracranial Aneurysms: A Retrospective Cohort Study” in Neurosurgery.^2^ Below, we present our point-by-point response to the Letter to the Editor.

## EXAMINATION OF ANEURYSM MORPHOLOGIES

The main reason for excluding fusiform intracranial aneurysms (IA) is that they are considered a distinct entity from saccular IAs. Fusiform IAs differ from saccular IAs in their morphology, pathogenesis, location, and epidemiology.^3^ Fusiform IAs are rare, comprising only around 4% of all IAs.^4^

While our focus on saccular IAs was driven by their higher prevalence and clinical significance, we acknowledge the importance of exploring potential associations with other morphologies. However, investigating nonsaccular IAs and their association with ankle-brachial index (ABI) levels would necessitate large-scale studies due to their rarity. Considering that fusiform IAs are associated with other cardiovascular diseases,^3^ there might also be an association with abnormal ABI levels.

## INTEROBSERVER RELIABILITY IN ABI MEASUREMENTS

The concern regarding interobserver variability of ABI measurements is indeed valid. In our study, ABI values were measured in a certified, busy angiolaboratory using high-quality equipment (laser-Doppler, Periflux 6000, Perimed AB). Our angiolaboratory routinely measures ABI and toe-brachial index (TBI) for approximately 1500 patients annually.

The issues raised regarding diverse equipment, learning curves, and techniques are crucial considerations, especially if ABI were to be widely applied as a screening tool for IAs. These concerns are particularly significant in underdeveloped countries or neurosurgery units lacking an active vascular unit within the hospital. We acknowledge that these aspects should have been discussed in further detail, along with the critical aspect of borderline ABI, which is highly sensitive to interobserver variability.

However, we believe that high-quality ABI measurements can be achieved with simple equipment after adequate training following generally accepted guidelines.^5,6^ The measurement of TBI, as mentioned, requires more expensive equipment and is technically more challenging. We are currently conducting an ongoing study, and a manuscript on the association between TBI and IAs is nearing submission. We believe that this forthcoming study will garner interest similar to the present article on ABI.

Furthermore, we are in the initial stages of planning a prospective cohort study inspired by the present article and our forthcoming manuscript containing TBI data. We aim to address the excellent comments provided by including them in the study setup and methodology.

Thank you once again for your valuable insights, which will undoubtedly contribute to the refinement and robustness of our future research endeavors.

## SELECTION BIAS

As stated in the Limitations section of our article, the criteria for performing cerebrovascular examination were not clearly defined. It is possible that patients with ischemic stroke, who are more likely to undergo angiographic studies, may have been overrepresented in our study. However, we believe this is unlikely to significantly affect our conclusions because our primary focus was on ABI levels, which are already known to be associated with stroke and other cardiovascular diseases.^7,8^ In addition, we had a comprehensive population of patients with normal ABI (n = 208) who underwent cerebrovascular imaging.

The question regarding the disparity in the impact of high ABI on the prevalence of unruptured IAs could be explained by several factors. In contrast to low ABI, high ABI is believed to be associated with medial arterial calcification and can occur in patients with conditions such as diabetes mellitus, medial calcinosis, or end-stage renal disease.^6^ Type 2 diabetes mellitus has not been shown to increase the risk of IAs.^9^ Furthermore, high ABI might be inversely associated with smoking,^10^ which is a known risk factor for IAs.^9^ Nevertheless, in our study,^2^ the prevalence of unruptured IAs was 5.3% (odds ratio 4.27; 95% CI, 0.83-22.00) in the high ABI group, and with a larger population, statistically significant differences might have been observed when compared with normal ABI.

## COMPREHENSIVE EVALUATION OF FOLLOW-UP ASSESSMENTS

The suggestion for a comprehensive evaluation of follow-up assessments to understand the longitudinal effects of ABI on IAs and its potential influence on rupture probability is well-received. Our pioneering study provides valuable insights that warrant further research into the relationship between ABI levels and the risk of IA rupture.
